# Evoked compound action potential (ECAP)-controlled closed-loop spinal cord stimulation in an experimental model of neuropathic pain in rats

**DOI:** 10.1186/s42234-023-00134-1

**Published:** 2024-01-10

**Authors:** Eline M. Versantvoort, Birte E. Dietz, Dave Mugan, Quoc C. Vuong, Saimir Luli, Ilona Obara

**Affiliations:** 1 School of Pharmacy, Newcastle University, Newcastle-Upon-Tyne, NE1 7RU UK; 2 Translational and Clinical Research Institute, Newcastle University, Newcastle-Upon-Tyne, NE1 7RU UK; 3 Saluda Medical Europe Ltd, Harrogate, HG2 8NB UK; 4 Bioscience Institute, Newcastle University, Newcastle-Upon-Tyne, NE1 7RU UK; 5 Preclinical In Vivo Imaging, Translational and Clinical Research Institute, Newcastle University, Newcastle-Upon-Tyne, NE2 4HH UK

**Keywords:** Evoked compound action potential, Rat, Closed-loop, Spinal cord stimulation, Neuropathic pain, In vivo electrophysiology

## Abstract

**Background:**

Preclinical models of spinal cord stimulation (SCS) are lacking objective measurements to inform translationally applicable SCS parameters. The evoked compound action potential (ECAP) represents a measure of dorsal column fiber activation. This measure approximates the onset of SCS-induced sensations in humans and provides effective analgesia when used with ECAP-controlled closed-loop (CL)-SCS systems. Therefore, ECAPs may provide an objective surrogate for SCS dose in preclinical models that may support better understanding of SCS mechanisms and further translations to the clinics. This study assessed, for the first time, the feasibility of recording ECAPs and applying ECAP-controlled CL-SCS in freely behaving rats subjected to an experimental model of neuropathic pain.

**Methods:**

Adult male Sprague–Dawley rats (200–300 g) were subjected to spared nerve injury (SNI). A custom-made six-contact lead was implanted epidurally covering T11-L3, as confirmed by computed tomography or X-ray. A specially designed multi-channel system was used to record ECAPs and to apply ECAP-controlled CL-SCS for 30 min at 50 Hz 200 µs. The responses of dorsal column fibers to SCS were characterized and sensitivity towards mechanical and cold stimuli were assessed to determine analgesic effects from ECAP-controlled CL-SCS. Comparisons between SNI rats and their controls as well as between stimulation parameters were made using omnibus analysis of variance (ANOVA) tests and *t*-tests*.*

**Results:**

The recorded ECAPs showed the characteristic triphasic morphology and the ECAP amplitude (mV) increased as higher currents (mA) were applied in both SNI animals and controls (SNI SCS-ON and sham SCS-ON). Importantly, the use of ECAP-based SCS dose, implemented in ECAP-controlled CL-SCS, significantly reduced mechanical and cold hypersensitivity in SNI SCS-ON animals through the constant and controlled activation of dorsal column fibers. An analysis of conduction velocities of the evoked signals confirmed the involvement of large, myelinated fibers.

**Conclusions:**

The use of ECAP-based SCS dose implemented in ECAP-controlled CL-SCS produced analgesia in animals subjected to an experimental model of neuropathic pain. This approach may offer a better method for translating SCS parameters between species that will improve understanding of the mechanisms of SCS action to further advance future clinical applications.

## Introduction

Spinal cord stimulation (SCS) is used as a safe and effective option for multiple chronic neuropathic pain disorders (Kumar et al. 2007; Kemler et al. 2008; Slangen et al. 2014). SCS has been shown to act via numerous physiological processes including regulation of neuroinflammatory responses (e.g., glial activation (Vallejo et al. 2016; Sato et al. 2014; Shu et al. 2020) and modulation of neurotransmitters (e.g., release of intracellular gamma-aminobutyric acid (GABA) or facilitation of descending inhibition involving serotoninergic mechanisms (Janssen et al. 2012; Song et al. 2009; Cui et al. 1997; Smits et al. 2012; Cui et al. 1996; Barchini et al. 2012). While different stimulation paradigms have been implemented into clinical practice (e.g., conventional, high-frequency, burst), it remains to be fully elucidated as to how these paradigms affect the spinal and supraspinal circuits (Smits et al. 2012; Joosten and Franken 2020; Jensen and Brownstone 2019). There is, however, an agreement that paresthesia-based SCS modulates pain processing via the activation of large, myelinated fibers in the dorsal column as confirmed by conduction velocity (CV) measurements (Dietz et al. 2022; Parker et al. 2012; Mekhail et al. 2020). Evoked compound action potentials (ECAP) have been successfully used as an objective measure to quantify the effect of SCS in terms of neural activation of dorsal column fibers, as they represent the summation of action potentials generated from the activated fibers (Parker et al. 2012). ECAPs have a triphasic morphology and their amplitude can be calculated from the difference between the first negative (N1) and second positive (P2) peaks (Parker et al. 2020; Parker et al. 2013; Parker et al. 2020). Once the ECAP threshold (ECAPT) has been reached, the ECAP amplitude has been shown to increase linearly with stimulation current and correlates with increasing intensity of sensation perceived by patients as paresthesia (Gmel et al. 2021). The perception threshold for stimulation-induced sensation in humans has been shown to coincide with the ECAPT (Gmel et al. 2021; Pilitsis et al. 2021) and the therapeutic ECAP target is set to a value between a patient’s perception threshold and the amplitude at which the sensations become uncomfortable (Parker et al. 2020). A major and unique advantage of ECAP recordings is that they can be incorporated into a closed-loop SCS system (ECAP-controlled CL-SCS) that allows for real-time adjustment of SCS dose to correct for variations in dorsal column activation that occur during posture alterations and physiological changes (Parker et al. 2020). Thus, in contrast to open-loop (OL)-SCS, ECAP-controlled CL-SCS can effectively control the SCS dose determined by the current delivered, resulting in a consistent level of dorsal column activation (Parker et al. 2020) and, importantly, has been proven superior to OL stimulation in human studies (Brooker et al. 2021; Mekhail et al. 2022).

SCS parameters used in rodent behavioral studies are based on motor reflexes (motor threshold, MT) elicited by stimulation (Smits et al. 2013) and/or by observing subtle changes in an animal's behavior that may suggest the onset of stimulation sensations (Koyama et al. 2018). Unlike MT, ECAPT has been shown to closely correlate with perception threshold in clinical settings (Gmel et al. 2021; Pilitsis et al. 2021). Thus, ECAP recordings provide an objective measure to assess the onset of stimulation-induced sensations, which can be utilized in preclinical SCS models. We recently demonstrated the feasibility of recording ECAPs from the dorsal column in naïve (non-neuropathic) anesthetized and freely behaving rats (Dietz et al. 2022), and others have achieved the same in naïve anesthetized animals (Parker et al. 2013; Parker et al. 2020; Cedeño et al. 2023). However, such recordings have not yet been used to assess ECAP-based therapy currently utilized in human patients for an animal model of neuropathic pain.

To address this gap, the aims of this study were twofold. Firstly, the responses of dorsal column fibers to SCS were characterized in rats subjected to an experimental model of neuropathic pain (spared nerve injury, SNI) and compared to uninjured controls. Secondly, the efficacy of ECAP-controlled CL-SCS in reducing mechanical and cold hypersensitivity was assessed in these animals. This study provides the first in vivo demonstration that the use of ECAP-based SCS dose implemented in ECAP-controlled CL-SCS produced analgesia in a preclinical model of neuropathic pain.

## Methods

### Animals

Adult male Sprague–Dawley rats (*n* = 45; 8–10-week-old; 200–300 g; Charles River Laboratories and Envigo) were acclimated to the colony room for at least 7 days after arrival and housed in groups of 2–3 animals per polyethylene cage (Comparative Biology Centre, Newcastle University, UK). After implantation of the SCS leads (described below) rats were housed in individual cages. Animals were maintained on a 12-h day-night cycle (lights on at 8:00 am; lights off at 8:00 pm) at controlled temperature (21 °C) and humidity (55%) and had ad libitum access to food and water*.* They were monitored throughout the study to ensure animal welfare. All experiments were performed under the UK Home Office license (P6694C943), with local approval from the Animal Welfare Ethical Review Body (AWERB), and in accordance with current United Kingdom legislation as defined in the Animals (Scientific Procedures) Act 1986. Additionally, the Animal Research: Reporting of In Vivo Experiments (ARRIVE) guidelines have been followed in reporting this study. Every effort was made to minimize animal suffering and to reduce the number of animals used in the study.

### Experimental design

**Fig. 1:**
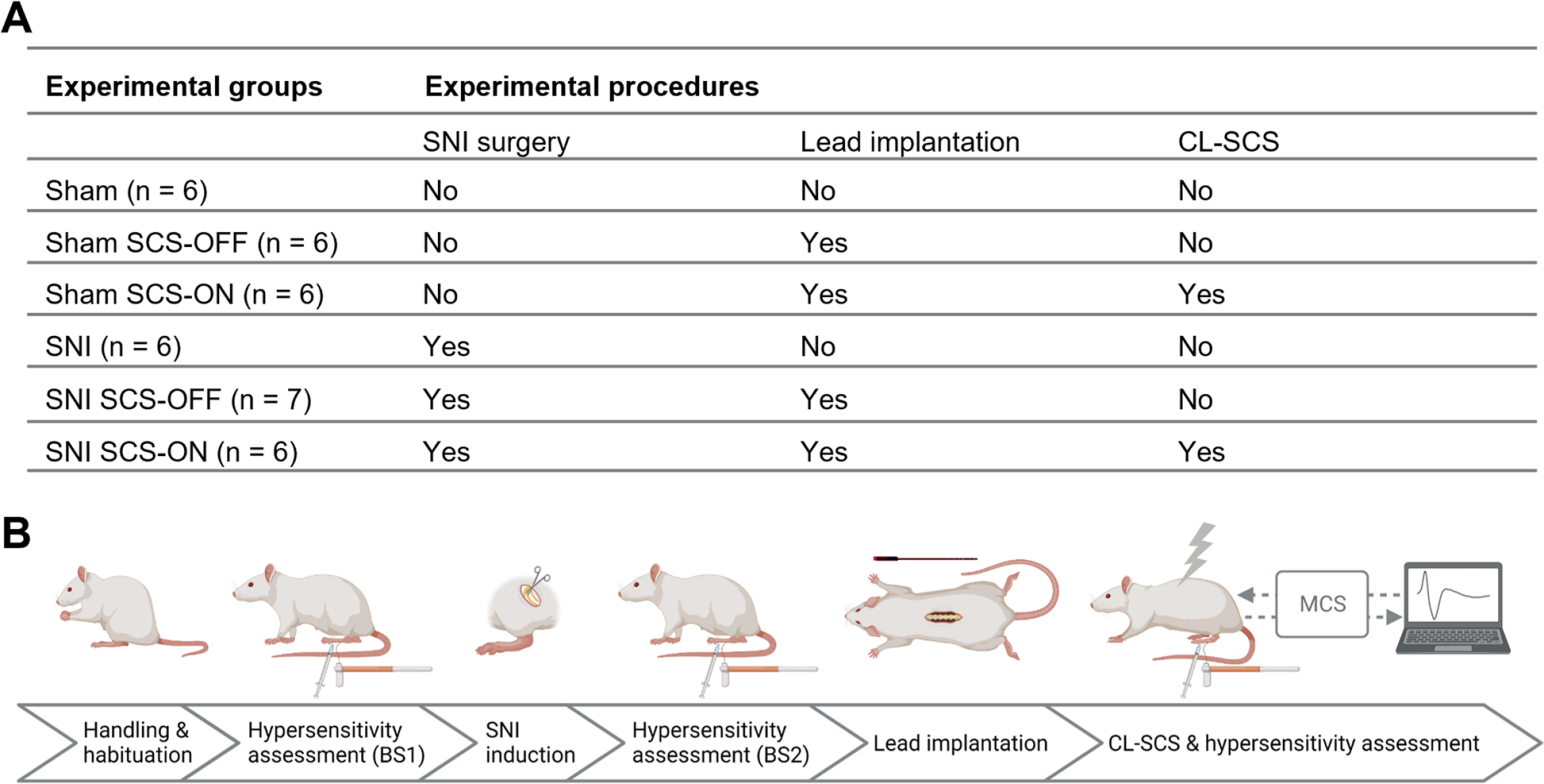
Schematic illustration of the experimental design and recording set-up. **A** The number of animals assigned to each experimental group. **B** Animals were handled and habituated to the test procedures prior to the experiments. Leads were implanted 8–15 days after pain induction and CL-SCS was administered approximately two days after lead implantation. Responses to mechanical and cold stimuli were assessed in all animals before and after each of the surgical procedures as well as in response to CL-SCS. BS1: baseline assessment of hypersensitivity before spared nerve injury (SNI) induction. BS2: baseline assessment of hypersensitivity before lead implantation. MCS: Multi-Channel-System MkII (Saluda Medical) used for stimulation and recordings, SCS-OFF: spinal cord stimulation off, SCS-ON: spinal cord stimulation on. *Created with BioRender.com*

The number of animals in the different conditions included in the final analyses of the experimental outcomes is shown in Fig. 1A.

All sham controls (*n* = 24) were not subjected to nerve injury. Six animals did not receive lead implantation (sham; Fig. 1A). The remaining 18 animals proceeded to lead implantation; however, 8 rats were excluded from the subsequent experimental steps and analyses due to technical problems associated with the signal-to-noise ratio of the data recordings. Out of the 10 animals, 6 animals were assigned to the sham SCS-OFF group and received no CL-SCS (Fig. 1A). The remaining four animals were subjected to CL-SCS (sham SCS-ON; Fig. 1A). In addition, two animals from the sham SCS-OFF group were subjected to CL-SCS one day after receiving no stimulation and therefore the sham SCS-ON group consisted of a total of 6 animals (Fig. 1A).

All SNI animals were subjected to nerve injury (*n* = 21). Six animals did not receive implantation or CL-SCS (SNI; Fig. 1A). The remaining 15 animals were implanted with SCS leads and received either no stimulation (SNI SCS-OFF; Fig. 1A) or CL-SCS (SNI SCS-ON; Fig. 1A). Two of the 8 animals in the SNI SCS-ON group were excluded from the analysis because their motor responses to stimulation were observed on the contralateral side instead of the side of injury.

The experimental steps are illustrated in Fig. 1B. Animals were handled and habituated to the test procedures prior to the experiments. Leads were implanted 8–15 days after pain induction and CL-SCS was administered approximately two days after lead implantation. Responses to mechanical and cold stimuli were assessed in all animals before and after each of the surgical procedures as well as in response to CL-SCS (sham SCS-ON and SNI SCS-ON). Animals that were not implanted with the leads (sham and SNI) or did not receive CL-SCS (sham SCS-OFF and SNI SCS-OFF) were assessed in the same time frame. Animals were randomly assigned to experimental groups up to and including the stage of lead implantation (Fig. 1B). The experimental set-up precluded randomization of animals to the SCS-OFF and SCS-ON groups. For practical reasons, assignment to the SCS-OFF and SCS-ON groups was based on the signal-to-noise ratios of the individual recordings obtained during input–output (IO) function collection. The experimental set-up prevented the behavioral tester from being blinded to the application of SCS (SCS-ON and SCS-OFF groups). To mitigate this limitation, a second behavioral observer confirmed the outcomes of animals’ responses. It should be also noted that this study did not aim to investigate the optimal stimulation intensity of CL-SCS in rats nor the efficacy of CL-SCS compared to OL-SCS, as has previously been investigated clinically (Mekhail et al. 2020; Mekhail et al. 2022). All pain hypersensitivity assessments and recordings were performed in freely behaving animals.

Stimulations and recordings were conducted with custom-made epidural leads with 6 channels (0.3 × 1.0 mm) equally spaced every 4 mm and connected to individual recording channels attached to the specially designed Multi-Channel System MKII (MCS; Saluda Medical) (Dietz et al. 2022; Parker et al. 2013). During stimulation, one channel was used for stimulation and recordings were made from the remaining five channels. The data acquisition was performed with custom software which controlled Data Acquisition (DAQ) units designed by United Electronic Industries with a sampling rate of 30 kHz and 24-bit analogue to digital converters. This set-up allowed for the continuous monitoring and real-time display of ECAPs during stimulation.

### Pain induction and hypersensitivity assessment

#### Pain induction

Neuropathic pain was induced using the SNI model (Decosterd and Woolf 2000). Animals underwent general anesthesia administered through a nose cone, 5% isoflurane with oxygen (flow rate of 2 L/min) as a carrier gas for induction, and 1.5–2.5% for maintenance. The procedure involved the transection of 2 of the 3 distal branches of the sciatic nerve, the common peroneal and tibial nerves, with 5.0 silk while leaving the sural nerve intact. The sham surgery procedure was identical except for the ligation and transection of the common peroneal and tibial nerves.

#### Pain hypersensitivity assessment

Mechanical hypersensitivity was determined using a series of von Frey filaments (bending forces 0.07, 0.16, 0.4, 0.6, 1.0, 1.4, 2.0, 4.0, 6.0, 8.0, 10.0, 15.0 and 26.0 g). Von Frey filaments with increasing force were applied to the lateral part of the hind paw, the sural territory, ipsilateral to the side of injury, starting with the filament of the lowest force (0.07 g), for five applications. The force of the von Frey filament that elicited an observed withdrawal response rate of 60% (3/5) was designated as the mechanical paw withdrawal threshold. 26.0 g bending force was defined as the cut-off value. To conform to Weber’s law and obtain a linear scale, thresholds were multiplied by 10,000 and logarithmically transformed (Mills et al. 2012).

For assessment of cold hypersensitivity, the acetone test was used (Choi et al. 1994). Cold hypersensitivity was tested via paw withdrawal latencies in response to a 50 µl drop of acetone. The drop of acetone was applied to the lateral plantar surface of the ipsilateral paw with a blunt needle connected to a syringe, avoiding mechanical stimulation of the paw. The withdrawal latency was defined as the time from the acetone application to the end of the observed withdrawal reflex (in seconds).

Prior to hypersensitivity assessment, animals were placed in a transparent box on an elevated mesh floor and were allowed to acclimatize to the experimental set-up for 30 min. Hypersensitivity assessment was conducted before SNI induction (BS1) as well as 8–15 days post-SNI (before lead implantation) to confirm the development of mechanical and cold hypersensitivity (BS2). Then, approximately two days after lead implantation, hypersensitivity assessment was performed before the start of CL-SCS (0 min), twice during CL-SCS (15 and 30 min) and twice after CL-SCS was terminated (45 and 60 min) to identify the effect of CL-SCS on mechanical and cold hypersensitivity. Animals were subjected first to the assessment of mechanical hypersensitivity, followed by the assessment of cold hypersensitivity.

### Electrophysiology

#### Lead implantation

Animals were implanted with custom-made epidural leads (Fig. 1A). Lead implantation was performed under general anesthesia administered via a nose cone (5% isoflurane with oxygen (flow rate of 2 L/min) as carrier gas used for induction; 1.5–2.5% for maintenance). A small laminectomy was performed at the level of T11 or T12, and subsequently, an SCS lead was inserted caudally into the epidural space such that active channels extended over spinal levels T11-L3, corresponding to the lower thoracic (T) and upper lumbar (L) vertebrae. The dura was kept intact during the procedure. The lead was fixed to the vertebrae caudal to the laminectomy plane using tissue glue. A second lead was placed subcutaneously and served as a reference and ground electrode (except in one animal where the reference and ground were at the same lead as the stimulation and recording channel). The wound was sutured in layers, and the proximal ends of the leads were tunneled subcutaneously to exit the skin at the base of the neck. The micro-contacts of both leads were connected to a cable that enabled connection to the MCS (Dietz et al. 2022). The lead position was confirmed by a computed tomography (CT) scan (SkyScan 1176, Bruker) or X-ray (Orange 1040HF, EcoRay). After lead implantation, animals were housed individually in separate cages. Animals received a mixture of bupivacaine and lidocaine during surgery (tunneled areas and incisions, lidocaine up to 10 mg/kg and bupivacaine up to 4 mg/kg) as well as enrofloxacin (10 mg/kg subcutaneously). Then, they received enrofloxacin (10 mg/kg subcutaneously) post-surgery for up to four days, once daily. Animals were allowed to recover for 1.80 ± 0.17 days prior to hypersensitivity assessment during CL-SCS.

#### IO function

Immediately before CL-SCS delivery, IO functions were collected from all animals that received stimulation (sham SCS-ON and SNI SCS-ON; Fig. 1A). During data collection, ECAP amplitudes (mV) in response to delivered currents (mA) were recorded. The ECAP amplitude is defined as the absolute difference between the N1 and P2 peaks. The channel covering T13, the level at which stimulation appears to provide more effective pain relief compared to more rostral levels (Smits et al. 2012), was used for stimulation. The stimulation current was increased in a stepwise manner from 0.0 mA until an ECAP could be observed on the recording screen, at the channel closest to the stimulation channel in the antidromic direction. Subsequently, the current intensity was increased until a motor response was observed in the mid-lower trunk or ipsilateral hind leg of the animal. IO functions relating ECAP amplitude to the stimulation current were obtained from all animals in the antidromic direction using 2 different sets of stimulation parameters for the stimulation frequency and pulse width (PW; 2 Hz 200 μs and 50 Hz 200 μs). The values for stimulation frequency and PW were based on previous preclinical studies in which 2 Hz 200 μs was used to determine MT (e.g., 10,31–34), while 50 Hz 200 μs was used to provide paresthesia-based stimulation (e.g., 6,8–10,31–34). These parameter settings allowed us to compare our ECAP-controlled CL-SCS results to previous work.

#### ECAPT and MT

Two methods were used to define ECAPT. First, extrapolated ECAPT was defined during the offline analysis of the data by a linear extrapolation of the IO function to 0-amplitude (Biesheuvel et al. 2018). The x-intercept was defined as ECAPT. Second, visually observed ECAPT was defined as the current at which the depolarization threshold was sufficient to generate a detectable ECAP at the channel closest to the stimulation channel in the antidromic direction, which was observed on the recording screen during the IO function collection at 50 Hz 200 μs (Dietz et al. 2022). In line with clinical practice, the visually observed ECAPT was used to set the stimulation intensity for the CL-SCS (described below), whereas the extrapolated ECAPT was used for the analyses. To minimize bias, visually observed ECAPTs were determined by two observers and the first offline detectable ECAP was determined by the same observer for all IO functions. MT was defined as the current that led to an observable motor response and was determined by the same observer throughout the experiment. The relationship between ECAPT (mA) and MT (mA) was established by measuring and comparing the relative magnitudes of these thresholds. Moreover, comparisons were made between sham and SNI animals across the different stimulation parameters.

#### CV

CV (meter per second (m/s)) is the speed at which an ECAP signal propagates along the neural pathway. CV was calculated in the antidromic direction by measuring ECAP propagation along the lead by stimulating the channel at T13 and measuring across the three channels positioned closest to the stimulation channel. The current was applied at 66–90% of MT, corresponding to the stimulation intensities used in previous experiments (Smits et al. 2013). CVs were based on the recordings made during the IO function collection.

#### CL-SCS

Recorded ECAPs were used to set stimulation intensity based on individually determined IO functions and were incorporated into a CL program which automatically adjusts current delivery pulse-on-pulse, i.e., 50 automated adjustments per second when programmed at 50 Hz, to maintain a constant level of dorsal column activation (Parker et al. 2020). The visually observed ECAPT (described above) recorded at the channel closest to the stimulation channel in the antidromic direction using 50 Hz 200 µs was used to determine stimulation intensity, which was set at a current above ECAPT which generated ECAPs large enough to maintain a constant level of dorsal column activation (CL). CL-SCS was delivered for 30 min at a frequency of 50 Hz and a PW of 200 µs. The channel covering T13 was used for stimulation and the remaining antidromic channels were used for the recording of ECAPs propagating caudally from the stimulation channel.

### Data analysis and statistics

Pre-processing and characterizing ECAPs were carried out using a custom-made toolbox for MATLAB (2013 release; Mathworks, Inc.) and custom scripts written in MATLAB (2022 release; Mathworks, Inc.).

Statistical analysis was performed using GraphPad Prism version 9.5.1 for Windows. All data are presented as mean ± standard error of the mean (SEM). The sample size was based on previously published studies and the validated nature of the tests (Curtis et al. 2018). In addition, the behavioral and electrophysiological experimental outcomes were assumed to be drawn from a normally distributed population (Gosselin 2019). Paired *t*-tests were used to compare ECAPTs with MTs. Omnibus analysis of variance (ANOVA) was used to investigate potential differences in IO function slope, ECAPT, MT, MT:ECAPT ratio and CV between sham SCS-ON and SNI SCS-ON animals as well as stimulation parameters. Additionally, omnibus analysis of variance (ANOVA) was conducted to assess the development of mechanical and cold hypersensitivity and the effect of CL-SCS. Significant interactions were followed by post-hoc *t*-tests with Bonferroni correction for multiple comparisons. Statistical significance was defined as *p* < 0.05 (corrected).

## Results

### Confirmation of pain hypersensitivity

The development of neuropathic pain, assessed by the measurement of mechanical and cold hypersensitivity, in all SNI animals was confirmed by comparing paw withdrawal thresholds (von Frey test) and paw withdrawal latencies (acetone test) between treatment groups over time. A two-way ANOVA investigating the effect of time (BS1; BS2; 0; 15; 30; 45 and 60 min) and treatment group (sham; sham SCS-OFF; sham SCS-ON; SNI; SNI SCS-OFF and SNI SCS-ON) on mechanical hypersensitivity showed a significant interaction between the two factors (*F*_(30, 186)_ = 31.418, *p* < 0.001). Similarly, a two-way ANOVA investigating the effect of time and treatment group on cold hypersensitivity showed a significant two-way interaction (*F*_(30, 186)_ = 4.805, *p* < 0.001). Post-hoc *t*-tests with Bonferroni correction revealed that a significant increase in response to mechanical stimuli was present after (BS2), but not before (BS1) SNI surgery in SNI animals when compared to their sham controls (sham vs. SNI: 5.01 ± 0.06 vs. 3.13 ± 0.08, *t*_(9.147)_ = 17.956, *p* < 0.001; sham SCS-OFF vs. SNI SCS-OFF: 5.11 ± 0.09 vs. 3.48 ± 0.14, *t*_(9.768)_ = 10.004, *p* < 0.001; sham SCS-ON vs. SNI SCS-ON: 5.23 ± 0.09 vs. 3.76 ± 0.21, *t*_(6.725)_ = 6.599, *p* = 0.005). Additionally, a significant increase in responses to cold stimuli was found in SNI animals after (BS2), but not before (BS1) SNI surgery when compared to their sham controls (sham vs. SNI: 0.18 ± 0.08 vs. 26.07 ± 2.07, *t*_(5.016)_ = 12.500, *p* < 0.001; sham SCS-OFF vs. SNI SCS-OFF: 0.01 ± 0.01 vs. 19.66 ± 0.87, *t*_(6.003)_ = 22.458, *p* < 0.001; sham SCS-ON vs. SNI SCS-ON: 0.00 ± 0.00 vs. 19.82 ± 2.17, *t*_(5.000)_ = 9.138, *p* = 0.004). These findings confirmed the development of mechanical and cold hypersensitivity in all SNI animals and not sham controls.

### Confirmation of the lead position in the spinal cord

**Fig. 2:**
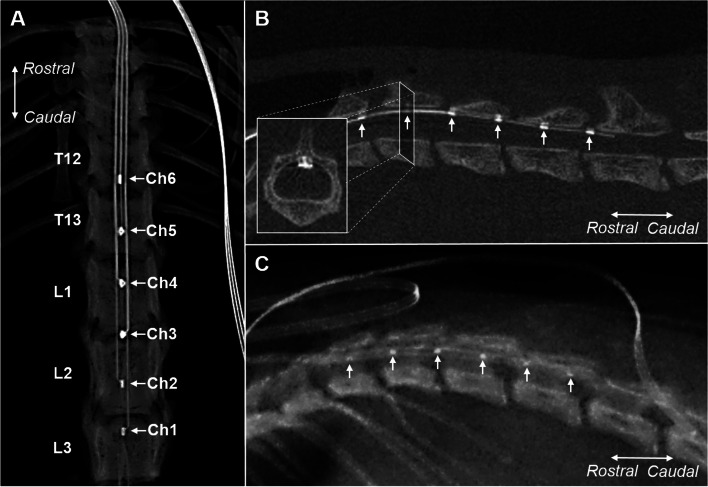
Examples of computed tomography (CT) and X-ray images confirming epidural implantation of the leads and position of the channels. Arrows indicate the 6 channels (Ch1-Ch6). **A** An example of a dorsal CT image confirming the area covered (T12-L3) by the epidural lead (SNI animal). **B** An example of a lateral CT image confirming the area covered (T12-L3) by the epidural lead. The inset shows a transverse section of the spinal cord, showing the position of the lead in the spinal epidural space (SNI animal). **C** An example of a lateral X-ray image confirming the area covered (T12-L2) by the epidural lead (sham animal). Ch: channel. SNI: spared nerve injury

CT or X-ray images confirmed the epidural implantation of the leads and the position of each channel in all animals (Fig. 2). Leads were inserted caudally in the dorsal epidural space at vertebrate levels T11 or T12, covering T11-L3 vertebrae.

### Characterization of ECAP recordings

**Fig. 3:**
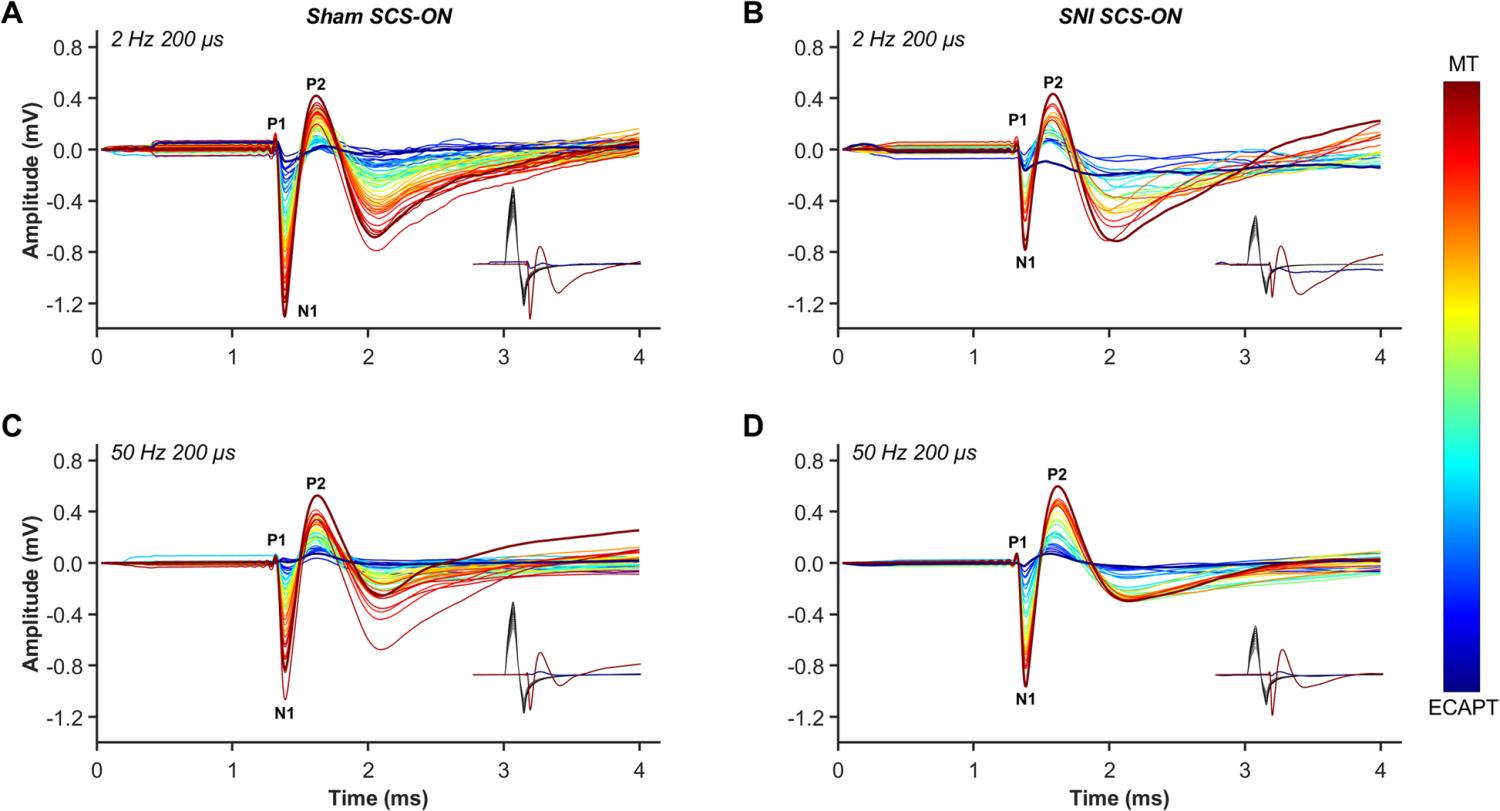
Examples of electrically evoked compound action potentials (ECAP) recorded from freely behaving sham SCS-ON (**A, C**) and SNI SCS-ON (**B, D**) animals at 2 Hz 200 µs and 50 Hz 200 µs from the first offline observable ECAP (ECAPT) to motor threshold (MT). The stimulation intensity (mA) from ECAPT to MT is indicated by the color bar. Recordings and measurements of ECAPs from (**A**) 0.067 mA to 0.121 mA (sham SCS-ON; 2 Hz 200 µs), (**B**) 0.029 mA to 0.051 mA (SNI SCS-ON; 2 Hz 200 µs), (**C**) 0.060 mA to 0.103 mA (sham SCS-ON; 50 Hz 200 µs), and (**D**) 0.024 mA to 0.049 mA (SNI SCS-ON; 50 Hz 200 µs). Recordings were captured from channel four (Ch4), the channel closest to the stimulation channel (Ch5, T13) in the antidromic (caudal) direction. The recorded neural signal consisted of a positive P1 peak followed by a negative N1 peak and a second positive P2 peak. The black traces in the insets indicate the different levels of applied stimulus intensity that resulted in the recorded neural signals (only recorded signals at the first offline observable ECAP and MT are illustrated in the inset). The ECAP signal was clearly distinguished from any signal recorded from the stimulation channel. SNI: spared nerve injury

ECAPs were recorded from all animals that received stimulation (sham SCS-ON and SNI SCS-ON; Fig. 1A). Examples of ECAPs recorded from freely behaving sham SCS-ON and SNI SCS-ON animals stimulated at 2 Hz 200 µs and 50 Hz 200 µs are illustrated in Fig. 3. As expected, the recorded ECAPs in all animals showed a characteristic triphasic morphology and the amplitude (P2-N1, mV) increased as higher currents (mA) were applied (Dietz et al. 2022; Parker et al. 2020; Parker et al. 2013). The N1 peaks, representing the time taken for the elicited ECAP to travel 4 mm from the stimulation channel (Ch5) to the recording channel (Ch4), were evoked between 1.373–1.433 ms (2 Hz 200 µs) and 1.373–1.453 ms (50 Hz 200 µs) in sham SCS-ON animals. Similarly, in SNI SCS-ON animals, the N1 peaks were evoked between 1.293–1.387 ms (2 Hz 200 µs) and 1.360–1.387 ms (50 Hz 200 µs). The ECAP signals were clearly distinguished from any signal recorded from the stimulation channel.

**Fig. 4:**
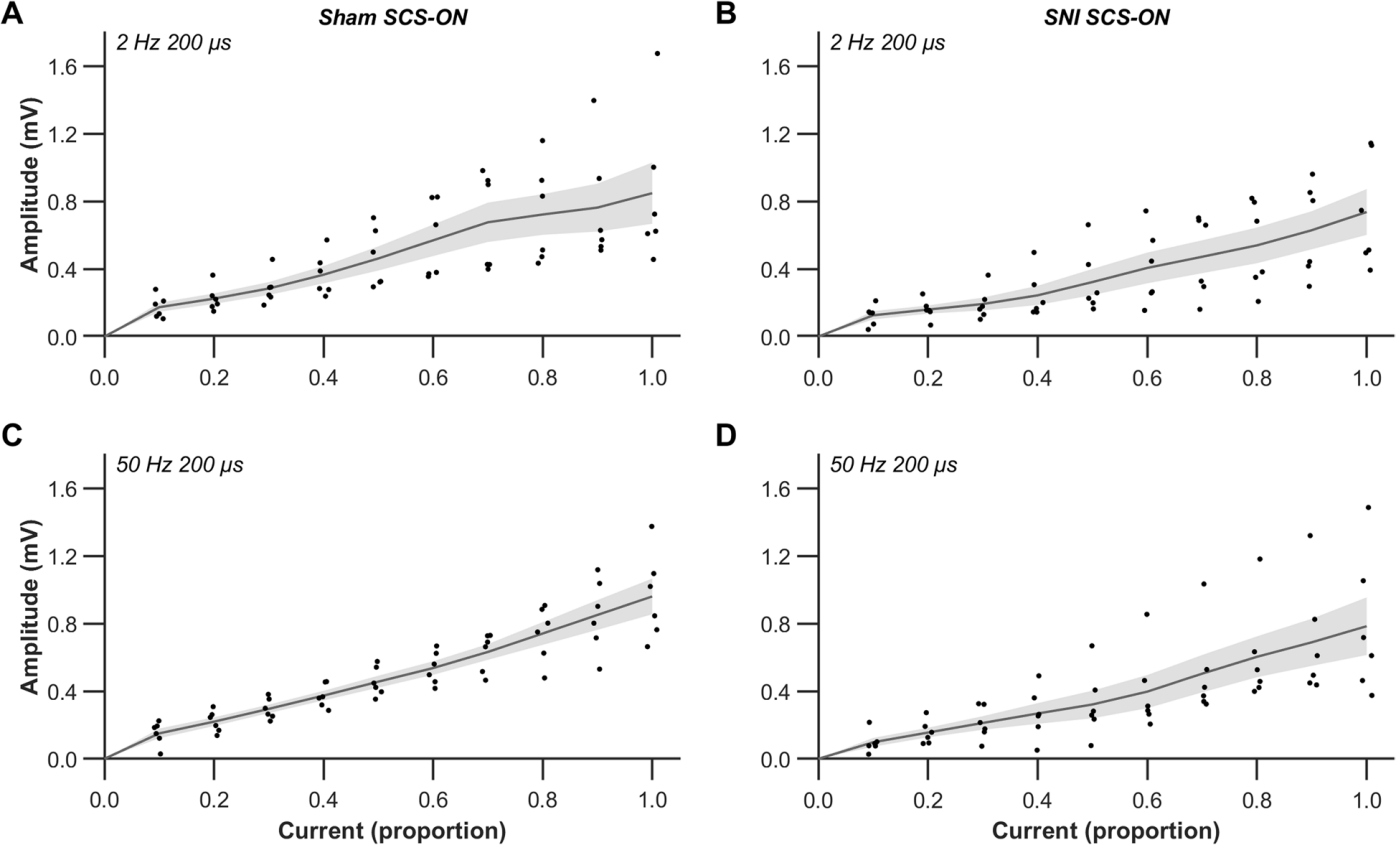
Input–output (IO) functions in sham SCS-ON (**A**, **C**) and SNI SCS-ON (**B**, **D**) animals using 2 Hz 200 µs and 50 Hz 200 µs. Evoked compound action potential (ECAP) recordings were taken from the channel closest to the stimulation channel (T13) in the antidromic direction. The current range of the interpolated function was scaled between 0.0 (extrapolated ECAP threshold) and 1.0 (motor threshold, MT). No significant differences in slope were found between sham SCS-ON and SNI SCS-ON animals (*p* = 0.663) nor between stimulation parameters (*p* = 0.975). The black dots represent data from individual animals, from the first offline observable ECAP to MT. The gray line and shaded region represent the mean ± SEM, n = 6. *p* < 0.05 (corrected) was used as the significance level (two-way ANOVA). SNI: spared nerve injury

IO functions were collected to identify the relationship between stimulation intensity and dorsal column activation in each animal. The IO functions represent offline analyzed recordings from the channel closest to the stimulation channel (T13) in the antidromic direction (Fig. 4). In two sham SCS-ON controls, stimulation was delivered at L1 rather than T13 throughout the experiment; however, this had no implications on the experimental outcomes. The raw data points, ranging from the first offline observable ECAP to MT, were interpolated with an assumption-free spline curve (smoothing parameter = 0.95) implemented in Matlab (2022 release; Mathworks, Inc). Linear regression was performed based on the interpolated data points previously obtained in Matlab and extrapolated ECAPTs were determined by linear extrapolation of IO functions to 0-amplitude. Subsequently, the current range of the interpolated function was scaled between 0.0 (extrapolated ECAPT) and 1.0 (MT). The growth of the ECAP amplitude (mV) with increasing current (mA) was linear from the first offline observable ECAP to the MT in all animals both at 2 Hz 200 µs (R^2^; sham: 0.88 ± 0.09 and SNI: 0.88 ± 0.06) and 50 Hz 200 µs (R^2^; sham: 0.98 ± 0.01 and SNI: 0.92 ± 0.03). The mean slopes in sham SCS-ON animals were 20.53 ± 3.13 mV/mA using 2 Hz 200 µs and 21.03 ± 2.45 mV/mA using 50 Hz 200 µs. In SNI SCS-ON animals, the mean slopes were 23.56 ± 6.37 mV/mA using 2 Hz 200 µs and 22.71 ± 7.60 mV/mA using 50 Hz 200 µs. No significant differences in slope were found between sham SCS-ON and SNI SCS-ON animals (*F*_(1, 20)_ = 0.195,* p* = 0.663) nor between stimulation parameters (*F*_(1, 20)_ = 0.001,* p* = 0.975).

### Comparisons of ECAPT, MT and CV across conditions

A two-way ANOVA indicated that the mean current required to generate ECAPT was significantly smaller in SNI SCS-ON animals when compared to sham SCS-ON controls (*F*_(1, 20)_ = 6.410,* p* = 0.020), but it did not differ between 2 Hz 200 μs and 50 Hz 200 μs (*F*_(1, 20)_ = 0.001,* p* = 0.976). In addition, a two-way ANOVA showed that MT currents did not differ significantly between sham SCS-ON and SNI SCS-ON animals (*F*_(1, 20)_ = 3.847,* p* = 0.064) nor between stimulation parameters (*F*_(1, 20)_ = 0.002, *p* = 0.964).

**Fig. 5:**
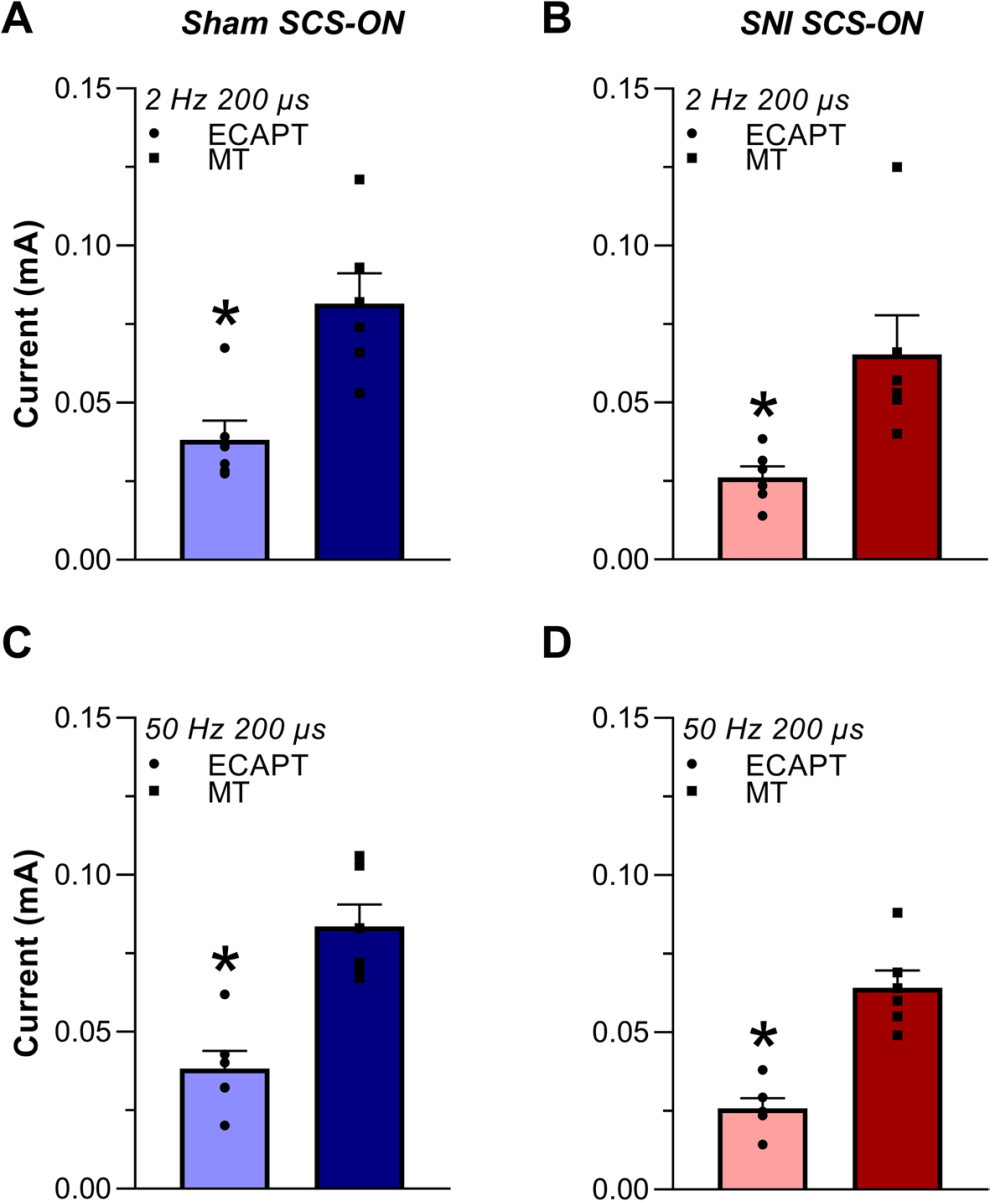
Mean current (mA) required to generate evoked compound action potential thresholds (ECAPT) and motor thresholds (MT) in freely behaving sham SCS-ON (**A**, **C**) and SNI SCS-ON (**B**, **D**) animals using 2 Hz 200 µs and 50 Hz 200 µs. **A** ECAPT in sham SCS-ON animals was 0.038 ± 0.006 mA and MT was 0.082 ± 0.010 mA using 2 Hz 200 µs. **B** ECAPT in SNI SCS-ON animals was 0.026 ± 0.004 mA and MT was 0.065 ± 0.012 mA using 2 Hz 200 µs. **C** ECAPT in sham SCS-ON animals was 0.038 ± 0.006 mA and MT was 0.084 ± 0.007 mA using 50 Hz 200 µs. **D** ECAPT in SNI SCS-ON animals was 0.026 ± 0.003 mA and MT was 0.064 ± 0.006 mA using 50 Hz 200 µs. Using both stimulation conditions, the current required to elicit MT was significantly higher than the current required to generate ECAPT in both sham SCS-ON (2 Hz 200 µs: *p* < 0.001; 50 Hz 200 µs: *p* < 0.001) and SNI SCS-ON (2 Hz 200 µs: *p* = 0.043; 50 Hz 200 µs: *p* < 0.001) animals. Stimulation was applied at the T13 vertebral level and recordings were measured antidromically to the stimulation channel. Data are presented as mean ± SEM, *n* = 6. *p* < 0.05 (corrected) was used as the significance level (paired t-test). * denotes significance compared to MT. SNI: spared nerve injury

A MT:ECAPT ratio was used to measure the relative current required to generate MT compared to the current required to generate ECAPT. Paired *t*-tests showed that, when using 2 Hz 200 μs stimulation, the current required to elicit MT was significantly higher than the current required to generate ECAPT (i.e., MT:ECAPT ratio > 1.0) in both sham SCS-ON (2.20 ± 0.15 times, *t*_(5)_ = 8.973, *p* < 0.001; Fig. 5A) and SNI SCS-ON (3.08 ± 1.19 times, t_(5)_ = 2.690, *p* = 0.043; Fig. 5B) animals. Similarly, when using 50 Hz 200 μs, the current to elicit MT was significantly higher than the current required to generate ECAPT in sham SCS-ON (2.32 ± 0.23 times, *t*_(5)_ = 11.884, *p* < 0.001; Fig. 5C) and SNI SCS-ON (2.63 ± 0.34 times, *t*_(5)_ = 8.975, *p* < 0.001; Fig. 5D) animals. Furthermore, a two-way ANOVA that analyzed the MT:ECAPT ratios showed no significant difference between sham SCS-ON and SNI SCS-ON animals (*F*_(1, 20)_ = 0.895, *p* = 0.356) nor between the different stimulation parameters (*F*_(1, 20)_ = 0.066, *p* = 0.800).

**Fig. 6:**
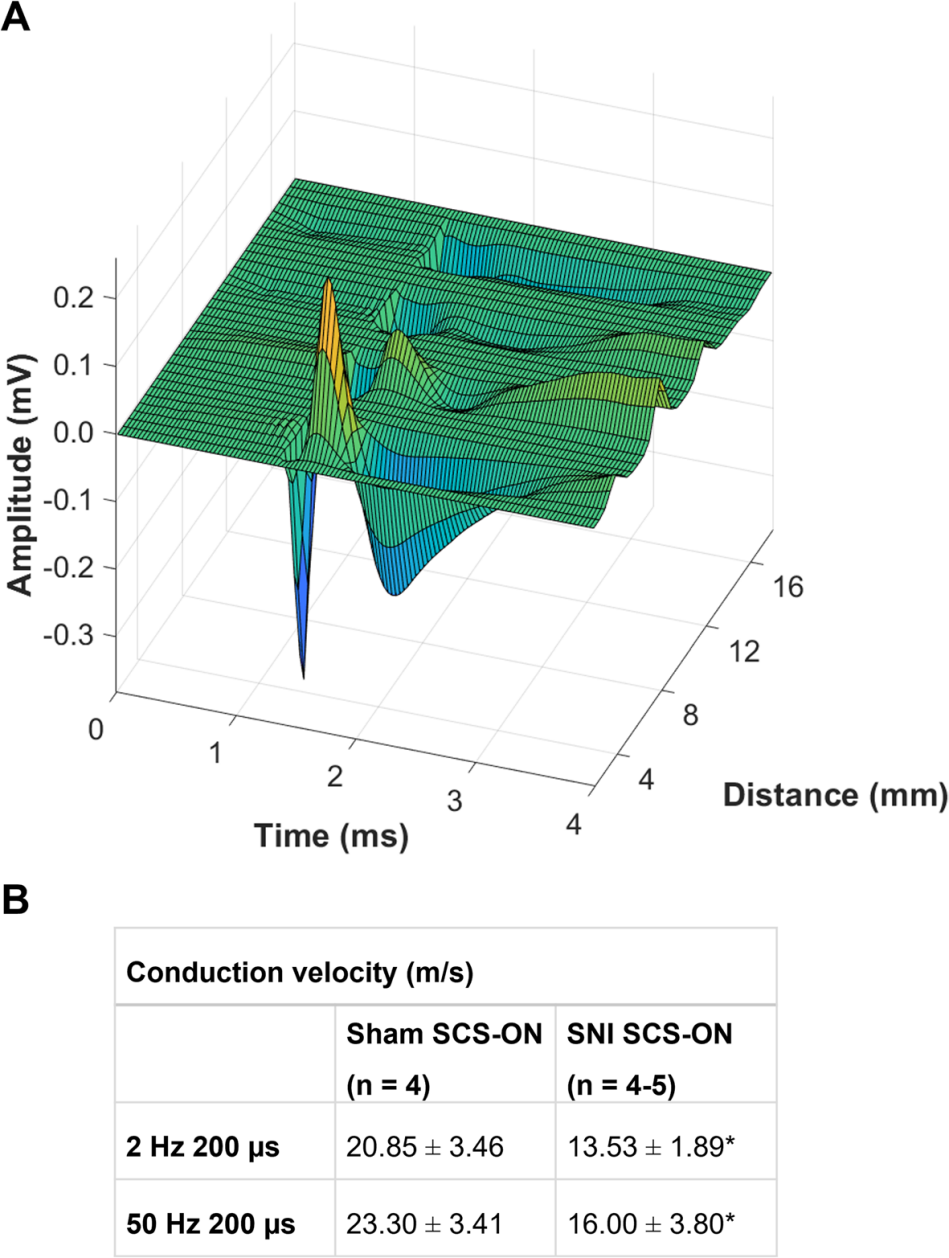
Propagating evoked compound action potential (ECAP) and mean conduction velocities (CV). **A** An example of an ECAP propagating over space and time in the antidromic direction (stimulation on channel 5 (T13), recording on channels four-one (4–16 mm from the stimulation channel; L1-L3) in an SNI SCS-ON animal. The distance between each of the channels on the leads is 4 mm. In this example, stimulation was applied at a current of 0.034 mA, corresponding to 69% of motor threshold (0.049 mA) and 1.4 times ECAP threshold (0.024 mA). ECAP amplitude decreased with increasing distance from the stimulation channel (4 mm: 0.63 mV, 8 mm: 0.20 mV, 12 mm: 0.09 mV, 16 mm: 0.08 mV). The speed at which the ECAP signal propagates along the neural pathway is defined as CV (measured in meter per second (m/s)). **B** Mean CVs in sham SCS-ON and SNI SCS-ON animals using 2 Hz 200 µs (*n* = 4 and 4, respectively) and 50 Hz 200 µs (*n* = 4 and 5, respectively). A significantly slower mean CV was found in SNI SCS-ON animals when compared to sham SCS-ON controls (*p* = 0.048). There was no effect of stimulation parameters on CV (*p* = 0.476). Stimulation was applied at T13, and recordings were obtained across the three channels positioned closest to the stimulation channel in the antidromic direction (T13-L2). Data are presented as mean ± SEM. *p* < 0.05 (corrected) was used as the significance level (two-way ANOVA). * denotes significance compared to sham animals. SNI: spared nerve injury

An example of an ECAP propagating over space and time, from channel four-one (4–16 mm from the stimulation channel; L1-L3), is shown in Fig. 6A. In this example, stimulation was applied on channel five (T13) at a current of 0.034 mA, corresponding to 69% of MT (0.049 mA) and 1.4 times ECAPT (0.024 mA). ECAP amplitude decreased with increasing distance from the stimulation channel in all animals (example shown in Fig. 6A; 4 mm: 0.63 mV, 8 mm: 0.20 mV, 12 mm: 0.09 mV, 16 mm: 0.08 mV). The mean CVs in the antidromic direction at a level of 66–90% MT are shown in Fig. 6B. Here, stimulation was applied at T13, and recordings were obtained across the three channels positioned closest to the stimulation channel in the antidromic direction (T13-L2). A significantly slower mean CV was found in SNI SCS-ON animals when compared to sham SCS-ON controls (*F*_(1, 13)_ = 4.762,* p* = 0.048), however, there was no difference between stimulation parameters observed (*F*_(1,13)_ = 0.540, *p* = 0.476).

### Assessment of the effect of CL-SCS on pain hypersensitivity

The visually observed ECAPT, which was estimated from observing the recording screen during the IO function collection at 50 Hz 200 µs, was used to determine the stimulation intensity for the CL-SCS. This threshold was 0.036 ± 0.007 mA in sham SCS-ON animals and 0.025 ± 0.002 mA in SNI SCS-ON animals. There was no significant difference between visually observed ECAPT and extrapolated ECAPT (described above) in both sham SCS-ON (*t*_(5)_ = 1.198, *p* = 0.285) and SNI SCS-ON (*t*_(5)_ = 0.233, *p* = 0.825) animals.

#### CL-SCS

**Fig. 7:**
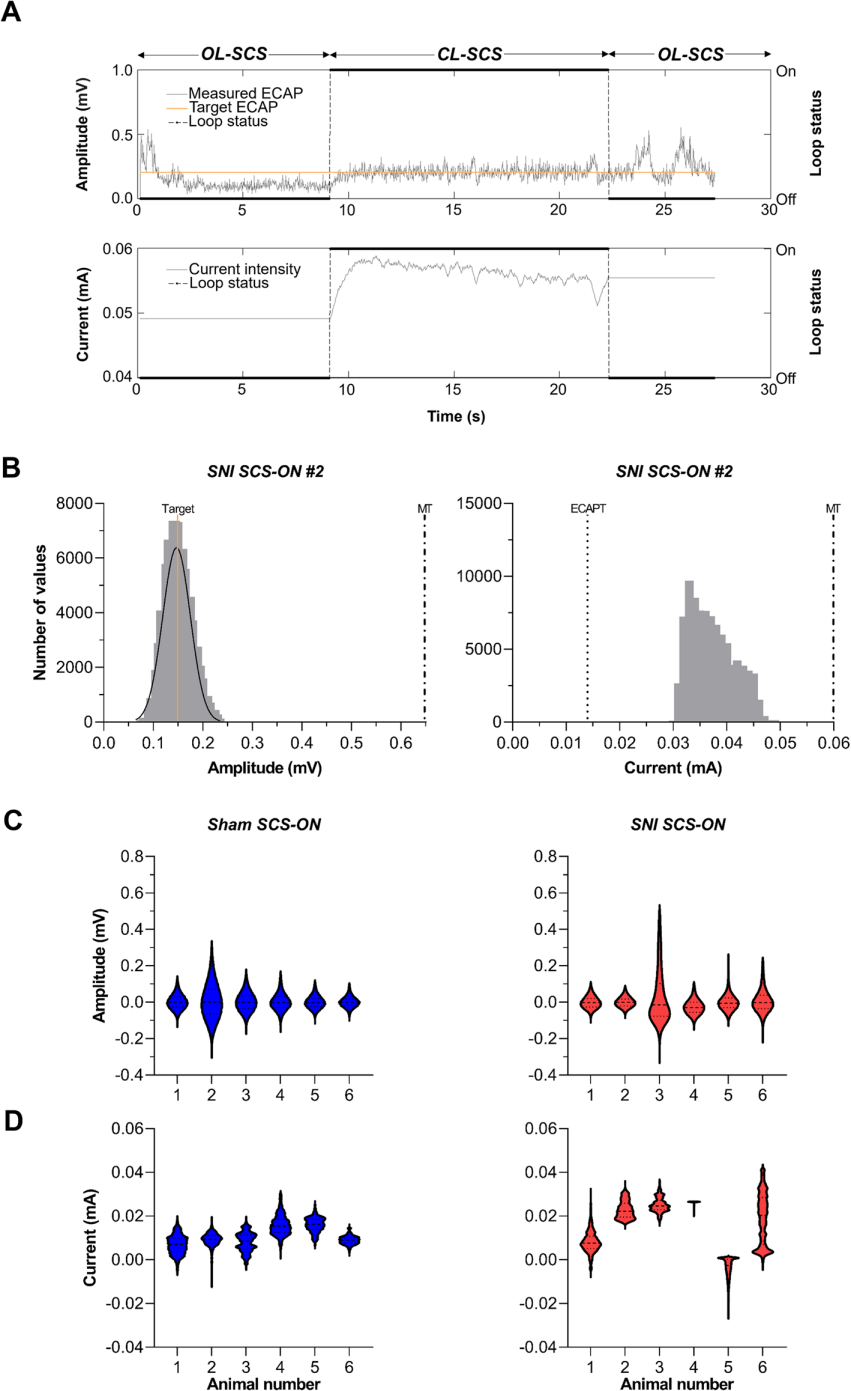
Application of closed-loop (CL)-SCS in freely behaving animals. **A** An example of open-loop (OL)-SCS vs. CL-SCS in a freely behaving SNI SCS-ON animal. **B** The frequency distribution of the measured evoked compound action potential (ECAP) amplitude and delivered current during 30 min of CL-SCS in an SNI SCS-ON animal (animal number two (#2)). The target amplitude was set at 0.1495 mV for this animal. The mean measured ECAP amplitude value was 0.1500 ± 0.0001 mV. Values followed a normal distribution around the target value (left panel). The current varied between 0.029 mA and 0.049 mA (right panel). The dash-dotted lines represent the motor threshold (MT; 0.6507 mV, 0.060 mA) and the dotted line represents the extrapolated ECAP threshold (ECAPT; 0.014 mA). **C** Frequency distributions of the difference between measured and target ECAP amplitude for each sham SCS-ON and SNI SCS-ON animal. **D** Frequency distributions of the difference between measured current and ECAPT for each sham SCS-ON and SNI SCS-ON animal. Current and amplitude values three standard deviations above or below the mean were considered outliers and were excluded from the analysis. SNI: spared nerve injury

ECAP-controlled CL-SCS was successfully applied in all SCS-ON animals. CL-SCS was applied on the channel covering T13 for 30 min at a stimulation intensity between ECAPT and MT that generated a robust ECAP recorded on the channel closest to the stimulation channel in the antidromic direction. An example of OL-SCS vs. CL-SCS during a 30 s period in an SNI SCS-ON animal is shown in Fig. 7A. During OL-SCS, the input current (mA) was kept constant, and the ECAP amplitude (mV) fluctuated. During CL-SCS, the input current was automatically adjusted to keep the ECAP amplitude more constant ensuring more consistent activation of the spinal cord.

The frequency distributions of the measured ECAP amplitude and delivered current during 30 min of CL-SCS in an SNI SCS-ON animal are presented (Fig. 7B). The ECAP target amplitude in this example was set at 0.1495 mV. The mean measured ECAP amplitude value was 0.1500 ± 0.0001 mV. Values followed a normal distribution around the target value (left panel). The current varied between 0.029 mA and 0.049 mA to keep the amplitude around the target value (right panel). During 30 min of 50 Hz CL-SCS, 89,914 ECAPs were recorded. Current and amplitude values three standard deviations above or below the mean were considered outliers and were excluded from the analysis for each animal. Frequency distributions of the difference between measured and target ECAP amplitude are shown for each animal (Fig. 7C). On average, the ECAP amplitude was at 22.18 ± 3.11% of MT (mean MT: 0.9500 mV) for sham SCS-ON animals and 33.69 ± 11.24% of the MT (mean MT: 0.7884 mV) for SNI SCS-ON animals. In addition, frequency distributions of the difference between measured current and ECAPT are presented for each animal (Fig. 7D).

#### ECAP-controlled CL-SCS and pain hypersensitivity

**Fig. 8:**
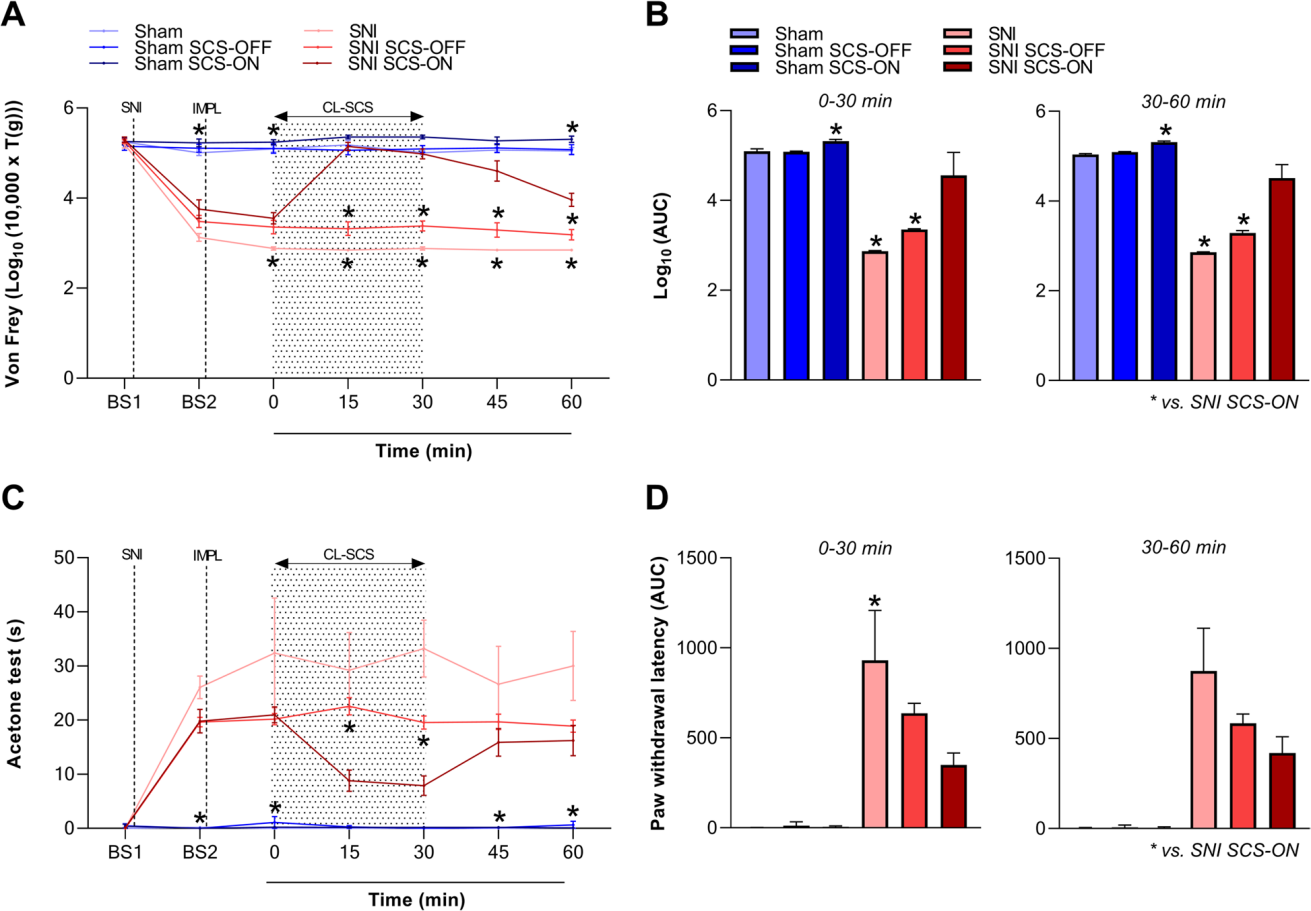
The effect of closed-loop spinal cord stimulation (CL-SCS) on mechanical (**A**, **B**) and cold (**C**, **D**) hypersensitivity was assessed using the von Frey and acetone tests. **A** Mean log10 thresholds (T, in grams) and (**C**) paw withdrawal latencies before SNI surgery (BS1), before lead implantation (BS2), and at 0, 15, 30, 45 and 60 min after the onset of CL-SCS delivery. The dotted lines represent spared nerve injury (SNI) surgery and lead implantation (IMPL). The pattern in the background represents the time that CL-SCS was delivered. **A** CL-SCS provided a significant reduction of mechanical hypersensitivity in the SNI SCS-ON animals when compared to the SNI (15 and 30 min: *p* < 0.001) and SNI SCS-OFF (15 and 30 min: *p* < 0.001) animals. **C** CL-SCS provided a significant reduction of cold hypersensitivity in the SNI SCS-ON animals when compared to the SNI SCS-OFF (15 min: *p* = 0.004, 30 min: *p* = 0.007) animals. **B**, **D** The area under the curve (AUC) for 0–30 min (left panel) and 30–60 min (right panel) time points, summarizing the measurements in (**A**) and (**C**). Data are presented as mean ± SEM, *n* = 6–7. *p* < 0.05 (corrected) was used as the significance level (one and two-way ANOVA, t-test). * denotes significance compared to SNI SCS-ON. Sham animals received no SNI surgery and no lead implantation (sham), or no stimulation (sham SCS-OFF) or were subjected to CL-SCS (sham SCS-ON). SNI animals received SNI surgery and no lead implantation (SNI), or no stimulation (SNI SCS-OFF) or were subjected to CL-SCS (SNI SCS-ON)

The effect of ECAP-controlled CL-SCS on mechanical and cold hypersensitivity was measured for a duration of 60 min that included 30 min of CL-SCS application (0–30 min) and 30 min when CL-SCS was terminated (30–60 min). As described above, a two-way ANOVA investigating the effect of time (BS1; BS2; 0; 15; 30; 45 and 60 min) and treatment group (sham; sham SCS-OFF; sham SCS-ON; SNI; SNI SCS-OFF and SNI SCS-ON) on mechanical hypersensitivity showed a significant interaction between the two factors (*F*_(30, 186)_ = 31.418, *p* < 0.001). Post-hoc *t*-tests with Bonferroni correction revealed that CL-SCS provided a significant reduction of mechanical hypersensitivity at 15 and 30 min in the SNI SCS-ON animals when compared to the SNI animals receiving no lead implantation (SNI; 15 min: *t*_(5.000)_ = 25.677, *p* < 0.001; 30 min: *t*_(6.015)_ = 17.821, *p* < 0.001) and the SNI SCS-OFF animals receiving no stimulation (SNI SCS-OFF; 15 min: *t*_(9.498)_ = 10.307, *p* < 0.001; 30 min: *t*_(10.937)_ = 10.009; Fig. 8A). In addition, two separate one-way ANOVAs investigating the von Frey area under the curve (AUC) during the application of CL-SCS (0–30 min) and after termination of CL-SCS (30–60 min) showed a significant effect of treatment group on von Frey AUC (0–30 min: *F*_(5, 31)_ = 71.957, *p* < 0.001; 30–60 min: *F*_(5, 31)_ = 53.891, *p* < 0.001; Fig. 8B). Bonferroni-corrected post-hoc *t*-tests revealed an increase in AUC in SNI SCS-ON animals when compared to SNI (0–30 min: *t*_(31)_ = 10.464, *p* < 0.001; 30–60 min: *t*_(31)_ = 8.298, *p* < 0.001) and SNI SCS-OFF (0–30 min: *t*_(31)_ = 8.010, *p* < 0.001; 30–60 min: *t*_(31)_ = 6.385, *p* < 0.001) animals both during and after CL-SCS.

There was a similar pattern of reduced pain sensitivity for the acetone test. A two-way ANOVA investigating the effect of time and treatment group on cold hypersensitivity showed a significant two-way interaction (*F*_(30, 186)_ = 4.805, *p* < 0.001). Cold hypersensitivity was significantly reduced in the SNI SCS-ON animals when compared to the SNI SCS-OFF animals (15 min: *t*_(10.147)_ = 5.421, *p* = 0.004; 30 min: *t*_(9.020)_ = 5.335, *p* = 0.007; Fig. 8C). For the AUC ANOVAs, a significant effect of treatment group on acetone AUC was observed during (*F*_(5, 31)_ = 11.262, *p* < 0.001) and after CL-SCS application (*F*_(5, 31)_ = 12.651, *p* < 0.001; Fig. 8D). A decrease in acetone AUC was found in SNI SCS-ON animals when compared to SNI animals during the application of CL-SCS (0–30 min: *t*_(31)_ = 3.483, *p* = 0.023).

## Discussion

This is the first study to characterize and utilize in vivo ECAP recordings from dorsal column fibers during SCS in freely behaving rats subjected to an experimental model of neuropathic pain. Importantly, our study provides the first successful application of ECAP-controlled CL-SCS resulting in significant reductions in mechanical and cold hypersensitivity induced by nerve injury, which is translationally comparable to the reduction in pain intensity reported in clinical subjects with neuropathic pain (Brooker et al. 2021; Mekhail et al. 2022). This CL-SCS effect was directly linked to the activation of large, myelinated fibers in the dorsal column as confirmed by epidural lead positions and the CV of the evoked signals. Together with our previous work (Dietz et al. 2022), the current findings may contribute to a better understanding of SCS mechanisms of action by introducing objective real-time measurements that are currently lacking in the SCS field.

In clinical trials of ECAP-controlled CL-SCS (Brooker et al. 2021; Mekhail et al. 2022), patients set the stimulation intensity to perceive a subjective comfortable sensation and that elicits a measurable ECAP. This intensity determined the ECAP target (i.e., the effective SCS dose). Law (1983) and Holsheimer (2002) further suggested that a stimulation intensity ≥ 1.3 times the intensity that generates a perceivable sensation is required for therapeutic benefit. As perception thresholds and ECAPTs have been shown to be relatively equivalent (Gmel et al. 2021; Pilitsis et al. 2021), the ECAPT can provide an objective measure to approximate sensation in non-verbal animals. Indeed, sensory threshold, defined as the intensity at which the animal became alert and adapted body posture or when visible disturbances of its behavior could be observed occurred when the stimulation intensities were around 40–50% of MT (Song et al. 2014; Shechter et al. 2013). In our study, the generated ECAPT was nearly half of the MT which most likely correlated with the sensory threshold. The use of evoked neurophysiological responses to inform an SCS dosage in preclinical models investigating SCS mechanisms has only been used during intra-operative SCS experiments (Yang et al. 2014; Yang et al. 2020; Guan et al. 2010). Our current findings demonstrate that ECAPT can be used to set the effective SCS dose in freely behaving rats. While this is an important step in the development of a preclinical model, it is unknown if the 1.3 factor proposed by Law (1983) and Holsheimer (2002) for clinical stimulation intensities is applicable to preclinical models and warrants further investigation.

Given the effectiveness of ECAP-controlled CL-SCS, we assessed the precision of the CL set-up using frequency histograms to analyze the ECAP-amplitude distribution (i.e., variance) around the ECAP target value (see Fig. 7C) and the corresponding stimulation-intensity distribution of stimulation intensities automatically selected by the system (see Fig. 7D). To aid comparison with clinical results, a range from ECAPT to MT was defined in our study to act as a translational surrogate for the therapeutic window previously described in a recent CL-SCS clinical study (Mekhail et al. 2022). In that study, ECAP-controlled CL-SCS demonstrated superior pain relief as compared to OL-SCS, as the CL system ensured greater and more consistent activation of dorsal column fibers. The variability in the dorsal column activation during OL-SCS, as compared to CL-SCS demonstrated in our animal model, matches that seen in clinical trials (e.g., see Fig. 4 in Mekhail et al*.* (2022)). While current preclinical SCS models use OL-SCS paradigms in freely behaving animals, potential variability in activation of dorsal column fibers, and therefore the SCS dose, is a factor that requires further research, particularly in relation to SCS paradigms that are presumed to be at sub-sensation threshold in the animal e.g., high-frequency or burst SCS (Zhai et al. 2022; Liao et al. 2020; Liao et al. 2020; Song et al. 2014; Chen et al. 2019). Importantly, in light of the current findings, ECAP-controlled CL-SCS can provide a valuable method to determine the optimal stimulation intensity, individualized for each animal, through the controlled activation of dorsal column fibers. This, in turn, can provide further insights into the existing discrepancies between preclinical and clinical results and inform ongoing debates regarding subcellular mechanisms of effect in SCS utilizing different stimulation paradigms (Meuwissen et al. 2018; Shechter et al. 2013; Cedeño et al. 2023).

Consistent with previous recordings in rats, sheep and humans (Dietz et al. 2022; Parker et al. 2020; Parker et al. 2013), ECAPs in this study showed the characteristic triphasic morphology in both SNI animals and their controls. As expected, the ECAP amplitude grew linearly with current intensity post ECAPT (Dietz et al. 2022; Parker et al. 2013; Sharma et al. 2023). However, a direct comparison of our work to that of recently published preclinical studies (Cedeño et al. 2023; Sharma et al. 2023) strongly suggests that multiple factors should be taken into careful consideration when analyzing ECAP recordings. Of particular significance is the position of the implanted leads for the stimulation and recording channels. We recorded ECAPs from equally spaced channels (4 mm) to control for distance whereas Cedano et al*.* (2023) recorded ECAPs from a separate lead positioned at an unreported distance from the stimulation contact. This resulted in more complex ECAP morphologies with nonlinear IO functions that were recorded from naïve anesthetized rats. In addition, Sharma et al*.* (2023) described triphasic ECAPs with a non-electromyography slow signal that was shown to be postsynaptic, again in naïve anesthetized rats, when recorded from two vertebral levels below the stimulation channel. The slow peaks described by Sharma et al*.* (2023) may also be present in our recordings (see Figs. 2B, C and 3B, C in Dietz et al*.* (2022)). Further characterization of these post-synaptic signals, particularly in relation to their presence, or absence, and possible impacts on analgesic effects would make an interesting line of future inquiry. As the latency between the application of the stimulation and the onset of the ECAP (N1) is a function of the distance between the stimulation and recording site, uncontrolled distance may result in varied outcomes even when using the same stimulation parameters. Moreover, it has been previously demonstrated in sheep and humans that ECAPs decrease in amplitude when recorded at increased distances from the stimulation channel (Parker et al. 2012; Parker et al. 2013; Parker et al. 2020). While this has also been observed in our studies (Dietz et al. 2022), future research is required to identify the nature of this phenomenon. Another significant factor to take into consideration when analyzing ECAP recordings in preclinical models is the morphometrics of the SCS lead. While Cedano et al*.* (2023) used cylindrical leads with a diameter of 0.72 mm and Sharma et al*.* (2023) used cylindrical leads with a diameter of 0.5 mm, our leads (0.2 mm thick with contacts 0.3 mm wide) are more akin to paddle leads used in humans and therefore will almost certainly have different stimulation characteristics in regard to activating structures in the spinal cord. This, however, warrants investigation and further scrutiny of the models employed in preclinical SCS research.

An unexpected finding of the current study was the observed differences in ECAPTs and CVs in SNI SCS-ON animals as compared to their sham SCS-ON controls. As stimulation intensities used in preclinical models are often reported as a percentage of MT (66–90%) (Smits et al. 2013), the relationship between ECAPT and MT was investigated in this study. During both 2 Hz and 50 Hz stimulation sessions, the current required to elicit MT was significantly higher than the current required to generate ECAPT in freely behaving sham SCS-ON and SNI SCS-ON animals, and the ratio was in line with our previously reported findings (Dietz et al. 2022). Interestingly, ECAPT was significantly lower in SNI SCS-ON animals compared to sham SCS-ON controls; however, MT:ECAPT ratio and MT were not significantly different. We also note that CVs were significantly slower in the SNI SCS-ON animals as compared to their sham SCS-ON controls. Reduced CVs have previously been shown in preclinical models of neuropathy (Hopkins and Gilliatt 1971) and are also known to occur in human peripheral neuropathies (Pietri et al. 1980). However, further research should be considered to investigate whether these results are replicable with larger sample sizes as these changes in spinal cord responses could provide information about the pathophysiology of nerve injury and the development and maintenance of neuropathic pain, as well as the underlying mechanisms of SCS.

## Conclusions

In summary, this study provides the first evidence that ECAP-controlled CL-SCS-induced analgesia in SNI rats is directly linked to the activation of large, myelinated dorsal column fibers. Implementing CL control in the rat model allows for better translation of preclinical SCS models through controlled and constant activation of dorsal column fibers. Future studies investigating dose–response relationships using ECAP-controlled CL-SCS can determine the optimal stimulation dose. Moreover, the efficacy of CL-SCS should be compared to other stimulation paradigms such as OL-SCS, and underlying mechanisms of action of SCS should be further investigated to improve clinical SCS applications.

## Data Availability

The datasets used and/or analyzed during the current study are available from the corresponding author on reasonable request.

## Abbreviations

ANOVA: Analysis of variance
ARRIVE: Animal Research: Reporting of In Vivo Experiments
AUC: Area under the curve
AWERB: Animal Welfare Ethical Review Body
BS: Baseline
Ch: Channel
CL: Closed-loop
CT: Computed tomography
CV: Conduction velocity
ECAP: Evoked compound action potential
ECAPT: Evoked compound action potential threshold
IMPL: Implantation
IO: Input-output
L: Lumbar
MCS: Multi-Channel-System
MT: Motor threshold
N1: First negative peak
OL: Open-loop
P2: Second positive peak
PW: Pulse width
SCS: Spinal cord stimulation
SCS-OFF: Spinal cord stimulation off
SCS-ON: Spinal cord stimulation on
SEM: Standard error of the mean
SNI: Spared nerve injury
T: Thoracic
